# State Prevalence and Ranks of Adolescent Substance Use: Implications for Cancer Prevention

**DOI:** 10.5888/pcd15.170345

**Published:** 2018-05-31

**Authors:** Jennifer L. Moss, Benmei Liu, Li Zhu

**Affiliations:** 1 National Cancer Institute, Bethesda, Maryland

## Abstract

**Introduction:**

This study statistically ranked states’ performance on adolescent substance use related to cancer risk (past-month cigarette smoking, binge alcohol drinking, and marijuana use).

**Methods:**

Data came from 69,200 adolescent participants (50 states and the District of Columbia) in the National Survey on Drug Use and Health (NSDUH) and 450,050 adolescent participants (47 states) in the Youth Risk Behavior Surveillance System (YRBSS). Adolescents were aged 14 to 17 years. For 2011–2015, we estimated and ranked states’ prevalence of adolescent substance use. We calculated the ranks’ 95% confidence intervals (CIs) using a Monte Carlo method with 100,000 simulations. Spearman correlations examined consistency of ranks.

**Results:**

Across states, the prevalence of cigarette smoking was 4.5% to 14.3% in NSDUH and 4.7% to 18.5% in YRBSS. Utah had the lowest prevalence (NSDUH: rank = 51 [95% CI, 47–51]; YRBSS: rank = 47 [95% CI, 46–47]), and states’ ranks across surveys were correlated (*r* = 0.66, *P* < .001). The prevalence of binge alcohol drinking was 5.9% to 14.3% (NSDUH) and 7.1% to 21.7% (YRBSS). Utah had the lowest prevalence (NSDUH: rank = 50 [95% CI, 40–51]; YRBSS: rank = 47 [95% CI, 47–47]), but ranks across surveys were weakly correlated (*r* = 0.38, *P* = .01). The prevalence of marijuana use was 6.3% to 18.7% (NSDUH) and 8.2% to 27.1% (YRBSS). Utah had the lowest prevalence of marijuana use (NSDUH: rank = 50 [95% CI = 33–51]; YRBSS: rank= 46 [95% CI, 46–46]), and ranks across surveys were correlated (*r* = 0.70, *P* < .001). Wide CIs for states ranked in the middle of each distribution obscured statistical differences among them.

**Conclusion:**

Variability emerged across adolescent substance use behaviors and surveys (perhaps because of administration differences). Most states showed statistically equivalent performance on adolescent substance use. Adolescents in all states would benefit from efforts to reduce substance use, to prevent against lifelong morbidity.

## Introduction

Substance use causes avoidable illness and death, including from cancer (1). Smoking tobacco causes lung, liver, and colorectal cancers, among others (2). Moderate to heavy alcohol consumption is associated with oropharyngeal, colorectal, and pancreatic cancers (3). Emerging evidence suggests a positive association between marijuana use and prostate and cervical cancer (4). Despite these risks, substance use is common: 60 million Americans smoke, 14 million are alcohol-dependent, and 14 million use illicit drugs (including marijuana) (1).

Reducing substance use among adolescents is particularly important for preventing cancer. First, lifelong substance use often begins in adolescence (5,6). For example, 88% of adult daily smokers began smoking before age 18 (7). Second, adolescence is a vulnerable period when people are particularly sensitive to substance use (2). Understanding adolescent substance use is therefore crucial to reducing the risk of related cancers.

Monitoring adolescent substance use, however, is challenging. Some adolescents may underreport use because of social desirability or fear of legal consequences (8) and others may overreport use to earn social cache from their peers (8). Studies comparing self-report to biometric measures of substance use have indicated that self-report measures have fair validity, with some adolescents underreporting and some overreporting use (8,9). However, quantifying the degree of uncertainty in estimates of adolescent substance use in surveillance surveys is important for leveraging these estimates for research and intervention purposes. Given that some public health efforts attempt to target adolescents in high-risk geographic areas, the ability to reliably identify which states have the highest or lowest prevalence of substance use may be called into question for several reasons: different surveillance surveys may identify different states; states likely rank differently across behaviors; and statistical uncertainty may undermine strong conclusions about differences across states. Understanding the extent of this problem has implications for surveillance research, specifically for cancer prevention.

We compared and ranked state estimates of past-month cigarette smoking, binge alcohol drinking, and marijuana use among adolescents from 2 population-based surveys: the National Survey of Drug Use and Health (NSDUH) and the Youth Risk Behavior Surveillance System (YRBSS). Both surveys collect substance use data for youths using cross-sectional, multistage probability sampling design, but each survey has its own strengths (10). A major strength of NSDUH is its ability to support estimates for all 50 states and the District of Columbia, and the data are collected every year. A major strength of YRBSS is its large state-level sample size; however, the survey is conducted every other year and not every state participates in the study or achieves adequate response rates. Comparison of the 2 surveys would highlight the consistencies or differences in rankings across different surveys. Calculation of confidence intervals (CIs) around ranks, often overlooked in ranking studies (11,12), allowed us to statistically evaluate consistencies and differences in state ranks within and across surveys and behaviors, and explore implications for cancer prevention.

## Methods

### Data sources and study populations

Data on adolescent substance use came from 2 population-based surveys: NSDUH (13) and YRBSS (14). NSDUH is an annual in-home survey sponsored by the Substance Abuse and Mental Health Services Administration (13) that estimates national- and state-level use of cigarettes, alcohol, and other drugs among people aged 12 years and older. Participants complete the NSDUH questionnaire on laptop computers assisted by trained interviewers (15). NSDUH publishes estimates of survey results annually (combining the current and previous years’ results) (13). For the current analysis, we used data from the 2011–2015 NSDUH surveys, which included 69,200 adolescents aged 14 to 17 years from 50 states and the District of Columbia (“states”); estimates of binge drinking included data only from survey years 2011–2014 (N = 58,000).

YRBSS is a biennial school-based survey coordinated by the Centers for Disease Control and Prevention (14) that monitors risk behaviors among adolescents. State education or health agencies administer surveys to representative samples of students in grades 9 through 12 (16). For states with response rates at or above 60%, YRBSS releases data for public use (16). For the current analysis, we used data from the 2011, 2013, and 2015 YRBSS surveys, which included 450,050 adolescents in grades 9 through 12 and aged 14 to 17 years from 47 states (excluding the District of Columbia, Minnesota, Oregon, and Washington, which either chose not to participate during all the survey years or did not achieve adequate response rates for public data release).

The current analysis was exempt from federal regulations for protections of human subjects because it involved secondary analysis of publicly available, de-identified data.

### Measures

From both data sources, we measured cigarette smoking, binge alcohol drinking, and marijuana use. Cigarette smoking was defined as smoking at least 1 cigarette in the previous 30 days. Binge alcohol drinking was defined as consuming 5 or more alcoholic drinks per drinking occasion on at least 1 of the previous 30 days. In 2015, NSDUH changed the definition of binge drinking among female respondents to 4 or more alcoholic drinks; thus, estimates of binge drinking from NSDUH include only 2011–2014 data for all participants. Marijuana use was defined as using marijuana at least once in the previous 30 days. Data on marijuana use in YRBSS were not available for Hawaii, so we excluded that state from analysis of this outcome.

In addition to substance use, we gathered data on adolescent sex (ie, male or female). Data on state of residence were collected as part of survey administration.

### Statistical analysis

First, we estimated the weighted percentage and standard error of each measure of substance use in each state and each survey among all adolescents and then stratified by sex. Sample weights and the complex survey design of NSDUH and YRBSS were incorporated in the estimation by using the PROC SURVEY procedures in SAS 9.3 software (SAS Institute, Inc). The standard errors were estimated by using the Taylor series linearization method (17). We also conducted paired samples *t* tests with Bonferroni adjustment to compare the substance use estimates for NSDUH versus YRBSS.

Next, we ranked states on their estimates for adolescent substance use from each survey. A Monte Carlo method generated simultaneous CIs around each state’s rankings using 100,000 simulations overall and by sex (separately for each survey) (12). Analyses assumed a normal distribution of estimates across states; preliminary analyses found that this assumption performed similarly to other distributional assumptions (including truncated normal, binomial, and lognormal). We determined each state’s median rank across the 100,000 simulations, rather than the raw rank generated from the estimates of substance use. Presenting median ranks maintains consistency between point estimates and 95% CIs (ie, all are estimated through the simulation analysis); in extreme cases (not observed in our data), the raw ranks could fall outside of the simulated 95% CI, but the median would not. The ranks did not necessarily span from 1 to 51 because of 1) ties and 2) the limited number of states available in YRBSS (maximum rank = 47 for cigarette smoking and binge alcohol drinking; 46 for marijuana use). We generated scatterplots of states’ ranks and CIs for each behavior and each survey.

Finally, we examined the consistency of rankings across surveys, behaviors, and subgroups (defined by adolescent sex) for states with data from both surveys by using Spearman rank correlation coefficients. All analyses used a criterion of *P* < .05 and were conducted in SAS 9.3 software.

## Results

### Cigarette smoking

**Figure 1:**
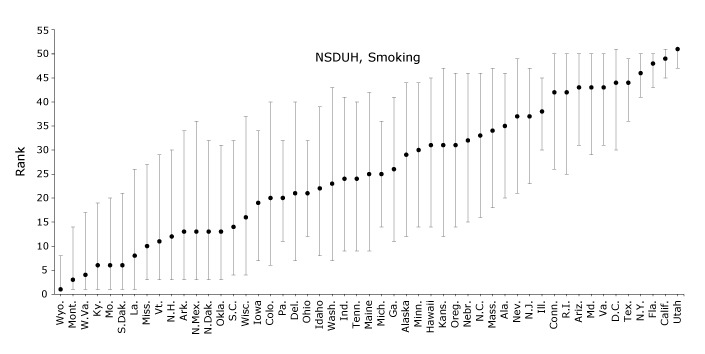

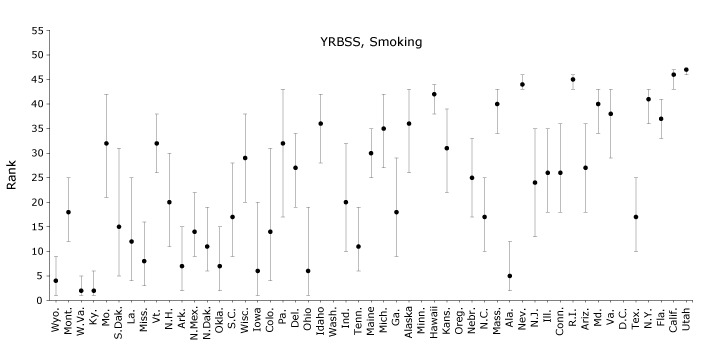
States’ simulated ranks for adolescent (aged 14–17 y) cigarette smoking as reported in the National Survey on Drug Use and Health (NSDUH) or Youth Risk Behavior Surveillance System (YRBSS), 2011–2015. YRBSS data were collected in 2011, 2013, and 2015; District of Columbia, Minnesota, Oregon, and Washington were excluded from YRBSS because they either chose not to participate or did not achieve adequate response rates. Past-month cigarette smoking was defined as smoking at least 1 cigarette in the previous 30 days. States are ordered by median rank in NSDUH across 100,000 simulations. Error bars indicate 95% confidence intervals. **Table:** 

In NSDUH, the prevalence of adolescent self-reported cigarette smoking ranged from 4.5% (standard error [SE], 0.9%) in Utah to 14.3% (SE, 1.3%) in Wyoming, with a median of 9.3% (Table 1). Wyoming was ranked 1 (95% CI, 1-7) and Utah 51 (95% CI, 47-51) in adolescent cigarette smoking (Figure 1). States in the middle of the distribution had particularly wide CIs; for example, Georgia was ranked 26 (95% CI, 11-41).

**Table 1:** Prevalence of Cigarette Smoking Among Adolescents Aged 14–17 Years and Simulated Ranking of States by Prevalence, National Survey on Drug Use and Health (NSDUH) and Youth Risk Behavior Surveillance System (YRBSS), 2011–2015

State	NSDUH		YRBSS	
	Rank^a^	% (SE)	Rank^a^	% (SE)
Alabama	35	8.0 (0.9)	5	16.7 (0.9)
Alaska	29	8.8 (1.0)	36	10.7 (0.8)
Arizona	43	6.9 (0.8)	27	12.1 (0.8)
Arkansas	13	10.6 (1.2)	7	16.2 (1.0)
California	49	5.7 (0.4)	46	6.5 (1.3)
Colorado	20	9.7 (1.2)	14	14.4 (1.5)
Connecticut	42	7.0 (1.0)	26	12.2 (0.8)
Delaware	21	9.6 (1.2)	27	12.2 (0.6)
District of Columbia	44	6.7 (1.0)	—	—
Florida	48	6.0 (0.5)	37	10.4 (0.4)
Georgia	26	9.1 (1.0)	18	13.7 (1.0)
Hawaii	31	8.5 (1.0)	42	9.4 (0.5)
Idaho	22	9.5 (1.0)	36	10.7 (0.6)
Illinois	38	7.6 (0.5)	26	12.2 (0.7)
Indiana	24	9.3 (1.1)	20	13.3 (1.0)
Iowa	19	9.9 (0.9)	6	16.3 (1.5)
Kansas	31	8.5 (1.2)	31	11.6 (0.7)
Kentucky	6	12.2 (1.2)	2	18.5 (1.0)
Louisiana	8	11.7 (1.3)	12	14.8 (1.2)
Maine	25	9.2 (1.1)	30	11.6 (0.4)
Maryland	43	6.9 (0.9)	40	10.0 (0.6)
Massachusetts	34	8.1 (1.0)	40	9.9 (0.5)
Michigan	25	9.2 (0.6)	35	10.8 (0.7)
Minnesota	30	8.6 (1.0)	—	—
Mississippi	10	11.1 (1.1)	8	15.8 (0.9)
Missouri	6	12.2 (1.2)	32	11.4 (0.9)
Montana	3	13.0 (1.3)	18	13.7 (0.6)
Nebraska	32	8.4 (1.0)	25	12.5 (0.7)
Nevada	37	7.7 (1.0)	44	7.9 (0.6)
New Hampshire	12	10.8 (1.1)	20	13.4 (0.8)
New Jersey	37	7.8 (0.8)	24	12.7 (1.0)
New Mexico	13	10.6 (1.4)	14	14.4 (0.7)
New York	46	6.3 (0.5)	41	9.6 (0.5)
North Carolina	33	8.3 (1.0)	17	13.8 (0.7)
North Dakota	13	10.7 (1.2)	11	15.0 (0.7)
Ohio	21	9.6 (0.6)	6	16.5 (1.6)
Oklahoma	13	10.7 (1.1)	7	16.1 (1.0)
Oregon	31	8.5 (1.1)	—	—
Pennsylvania	20	9.7 (0.6)	32	11.3 (1.3)
Rhode Island	42	7.1 (1.1)	45	7.6 (0.7)
South Carolina	14	10.5 (1.1)	17	13.9 (0.9)
South Dakota	6	12.2 (1.3)	15	14.2 (1.4)
Tennessee	24	9.3 (1.0)	11	15.0 (0.8)
Texas	44	6.8 (0.5)	17	13.9 (0.7)
Utah	51	4.5 (0.9)	47	4.7 (0.4)
Vermont	11	10.9 (1.1)	32	11.3 (0.5)
Virginia	43	6.9 (0.8)	38	10.3 (0.8)
Washington	23	9.4 (1.3)	—	—
West Virginia	4	12.6 (1.3)	2	18.4 (0.8)
Wisconsin	16	10.2 (1.2)	29	11.8 (0.7)
Wyoming	1	14.3 (1.3)	4	17.1 (0.8)

Abbreviation: SE, standard error.

a Ranks are the median rank generated in 100,000 Monte Carlo simulations. Past-month cigarette smoking was defined as smoking at least 1 cigarette in the previous 30 days. YRBSS data were collected in 2011, 2013, and 2015; District of Columbia, Minnesota, Oregon, and Washington were excluded from YRBSS because they either chose not to participate or did not achieve adequate response rates.

State	NSDUH Rank (95% CI)	YRBSS Rank (95% CI)
Wyoming	1 (1, 8)	4 (1, 9)
Montana	3 (1, 14)	18 (12, 25)
West Virginia	4 (1, 17)	2 (1, 5)
Kentucky	6 (1, 19)	2 (1, 6)
Missouri	6 (1, 20)	32 (21, 42)
South Dakota	6 (1, 21)	15 (5, 31)
Louisiana	8 (1, 26)	12 (4, 25)
Mississippi	10 (3, 27)	8 (3, 16)
Vermont	11 (3, 29)	32 (26, 38)
New Hampshire	12 (3, 30)	20 (11, 30)
Arkansas	13 (3, 34)	7 (2, 15)
New Mexico	13 (3, 36)	14 (9, 22)
North Dakota	13 (3, 32)	11 (6, 19)
Oklahoma	13 (3, 31)	7 (2, 15)
South Carolina	14 (4, 32)	17 (9, 28)
Wisconsin	16 (4, 37)	29 (20, 38)
Iowa	19 (7, 34)	6 (1, 20)
Colorado	20 (6, 40)	14 (4, 31)
Pennsylvania	20 (11, 32)	32 (17, 43)
Delaware	21 (7, 40)	27 (19, 34)
Ohio	21 (12, 32)	6 (1, 19)
Idaho	22 (8, 39)	36 (28, 42)
Washington State	23 (7, 43)	–
Indiana	24 (9, 41)	20 (10, 32)
Tennessee	24 (9, 40)	11 (6, 19)
Maine	25 (9, 42)	30 (25, 35)
Michigan	25 (14, 36)	35 (27, 42)
Georgia	26 (11, 41)	18 (9, 29)
Alaska	29 (12, 44)	36 (26, 43)
Minnesota	30 (14, 44)	–
Hawaii	31 (14, 45)	42 (38, 44)
Kansas	31 (12, 47)	31 (22, 39)
Oregon	31 (14, 46)	–
Nebraska	32 (15, 46)	25 (17, 33)
North Carolina	33 (16, 46)	17 (10, 25)
Massachusetts	34 (18, 47)	40 (34, 43)
Alabama	35 (20, 46)	5 (2, 12)
Nevada	37 (21, 49)	44 (43, 46)
New Jersey	37 (23, 47)	24 (13, 35)
Illinois	38 (30, 45)	26 (18, 35)
Connecticut	42 (26, 50)	26 (18, 36)
Rhode Island	42 (25, 50)	45 (43, 46)
Arizona	43 (31, 50)	27 (18, 36)
Maryland	43 (29, 50)	40 (34, 43)
Virginia	43 (31, 50)	38 (29, 43)
Washington DC	44 (30, 51)	–
Texas	44 (36, 49)	17 (10, 25)
New York	46 (41, 50)	41 (36, 43)
Florida	48 (43, 50)	37 (33, 41)
California	49 (45, 51)	46 (43, 47)
Utah	51 (47, 51)	47 (46, 47)

In YRBSS, the prevalence of adolescent cigarette smoking ranged from 4.7% (SE, 0.4%) in Utah to 18.5% (SE, 1.0%) in Kentucky, with a median of 12.5% (Table 1). In paired samples *t* tests, rates of cigarette smoking were higher in YRBSS than in NSDUH across states (*P* < .001). Kentucky was ranked 2 (95% CI, 1-6) and Utah 47 (95% CI, 46-47) in cigarette smoking (Figure 1). The ranks derived from NSDUH were not necessarily equal to those from YRBSS, but they were correlated (*r* = 0.64, *P* < .001).

### Binge alcohol drinking

**Figure 2:**
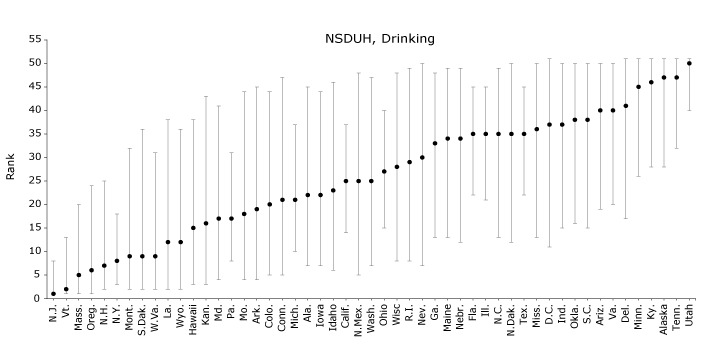

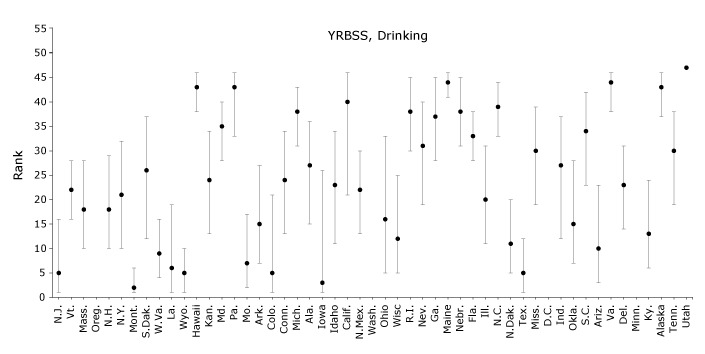
States’ simulated ranks for adolescent (aged 14–17 y) alcohol consumption as reported in the National Survey on Drug Use and Health (NSDUH) or Youth Risk Behavior Surveillance System (YRBSS), 2011–2015. YRBSS data were collected in 2011, 2013, and 2015; District of Columbia, Minnesota, Oregon, and Washington were excluded from YRBSS because they either chose not to participate or did not achieve adequate response rates. Past-month binge drinking was defined as consuming 5 or more alcoholic drinks per drinking occasion on at least 1 of the previous 30 days. NSDUH data on binge alcohol drinking came from survey years 2011–2014 only. States are ordered by median rank in NSDUH across 100,000 simulations. Error bars indicate 95% confidence intervals. **Table:** 

In NSDUH, the prevalence of adolescent self-reported binge alcohol drinking ranged from 5.9% (SE, 1.1%) in Utah to 14.3% (SE, 1.4%) in New Jersey, with a median of 9.4% (Table 2). New Jersey was ranked 1 (95% CI, 1-8) and Utah 50 (95% CI, 40-51) in adolescent binge alcohol drinking (Figure 2). States in the middle of the distribution had wide CIs; for example, New Mexico was ranked 25 (95% CI, 5-48).

**Table 2:** Prevalence of Binge Drinking Among Adolescents Aged 14–17 Years and Simulated Ranking of States by Prevalence, National Survey on Drug Use and Health (NSDUH) and Youth Risk Behavior Surveillance System (YRBSS), 2011–2015

State	NSDUH		YRBSS	
	Rank^a^	% (SE)	Rank^a^	% (SE)
Alabama	22	9.7 (1.1)	27	16.5 (0.9)
Alaska	47	7.0 (1.1)	43	13.2 (0.8)
Arizona	40	8.1 (1.0)	10	19.2 (1.1)
Arkansas	19	10.1 (1.4)	15	18.2 (0.9)
California	25	9.5 (0.5)	40	14.0 (1.7)
Colorado	20	10.0 (1.2)	5	20.7 (1.8)
Connecticut	21	9.8 (1.4)	24	17.0 (0.8)
Delaware	41	8.0 (1.2)	23	17.2 (0.6)
District of Columbia	37	8.4 (1.4)	—	—
Florida	35	8.6 (0.5)	33	15.4 (0.4)
Georgia	33	8.8 (1.0)	37	14.5 (1.0)
Hawaii	15	10.6 (1.2)	43	13.1 (0.6)
Idaho	23	9.6 (1.2)	23	17.1 (1.0)
Illinois	35	8.6 (0.6)	20	17.5 (0.8)
Indiana	37	8.4 (1.1)	27	16.6 (1.1)
Iowa	22	9.7 (1.1)	3	21.3 (2.4)
Kansas	16	10.4 (1.4)	24	17.0 (0.9)
Kentucky	46	7.3 (1.0)	13	18.6 (0.8)
Louisiana	12	11.0 (1.4)	6	20.5 (1.5)
Maine	34	8.7 (1.1)	44	12.8 (0.4)
Maryland	17	10.3 (1.2)	35	15.1 (0.6)
Massachusetts	5	12.3 (1.2)	18	17.8 (0.7)
Michigan	21	9.8 (0.7)	38	14.4 (0.7)
Minnesota	45	7.5 (0.9)	—	—
Mississippi	36	8.5 (1.2)	30	15.9 (0.9)
Missouri	18	10.2 (1.4)	7	20.0 (1.1)
Montana	9	11.5 (1.4)	2	21.7 (0.5)
Nebraska	34	8.7 (1.2)	38	14.3 (0.8)
Nevada	30	9.0 (1.5)	31	15.7 (1.0)
New Hampshire	7	11.8 (1.2)	18	17.8 (0.8)
New Jersey	1	14.3 (1.4)	5	20.8 (1.4)
New Mexico	25	9.5 (1.4)	22	17.2 (0.7)
New York	8	11.7 (0.8)	21	17.3 (0.9)
North Carolina	35	8.6 (1.1)	39	14.1 (0.7)
North Dakota	35	8.6 (1.2)	11	18.9 (0.8)
Ohio	27	9.3 (0.6)	16	18.0 (1.3)
Oklahoma	38	8.3 (1.1)	15	18.2 (0.9)
Oregon	6	12.1 (1.3)	—	—
Pennsylvania	17	10.3 (0.7)	43	13.2 (1.1)
Rhode Island	29	9.1 (1.3)	38	14.4 (0.9)
South Carolina	38	8.3 (1.2)	34	15.2 (1.0)
South Dakota	9	11.4 (1.5)	26	16.7 (1.0)
Tennessee	47	7.0 (0.9)	30	16.1 (0.8)
Texas	35	8.6 (0.6)	5	20.5 (0.9)
Utah	50	5.9 (1.1)	47	7.1 (0.7)
Vermont	2	13.6 (1.4)	22	17.3 (0.4)
Virginia	40	8.1 (0.9)	44	13.0 (0.7)
Washington	25	9.5 (1.2)	—	—
West Virginia	9	11.4 (1.2)	9	19.5 (0.8)
Wisconsin	28	9.2 (1.2)	12	18.7 (1.0)
Wyoming	12	11.0 (1.3)	5	20.8 (0.7)

Abbreviation: SE, standard error.

a Ranks are the median rank generated in 100,000 Monte Carlo simulations. Past-month binge drinking was defined as consuming 5 or more alcoholic drinks per drinking occasion on at least 1 of the previous 30 days. NSDUH data on binge alcohol drinking came from survey years 2011–2014 only. YRBSS data were collected in 2011, 2013, and 2015; District of Columbia, Minnesota, Oregon, and Washington were excluded from YRBSS because they either chose not to participate or did not achieve adequate response rates.

State	NSDUH Rank (95% CI)	YRBSS Rank (95% CI)
New Jersey	1 (1, 8)	5 (1, 16)
Vermont	2 (1, 13)	22 (16, 28)
Massachusetts	5 (1, 20)	18 (10, 28)
Oregon	6 (1, 24)	–
New Hampshire	7 (2, 25)	18 (10, 29)
New York	8 (3, 18)	21 (10, 32)
Montana	9 (2, 32)	2 (1, 6)
South Dakota	9 (2, 36)	26 (12, 37)
West Virginia	9 (2, 31)	9 (4, 16)
Louisiana	12 (2, 38)	6 (1, 19)
Wyoming	12 (2, 36)	5 (1, 10)
Hawaii	15 (3, 38)	43 (38, 46)
Kansas	16 (3, 43)	24 (13, 34)
Maryland	17 (4, 41)	35 (28, 40)
Pennsylvania	17 (8, 31)	43 (33, 46)
Missouri	18 (4, 44)	7 (2, 17)
Arkansas	19 (4, 45)	15 (7, 27)
Colorado	20 (5, 44)	5 (1, 21)
Connecticut	21 (5, 47)	24 (13, 34)
Michigan	21 (10, 37)	38 (31, 43)
Alabama	22 (7, 45)	27 (15, 36)
Iowa	22 (7, 44)	3 (1, 26)
Idaho	23 (6, 46)	23 (11, 34)
California	25 (14, 37)	40 (21, 46)
New Mexico	25 (5, 48)	22 (13, 30)
Washington State	25 (7, 47)	–
Ohio	27 (15, 40)	16 (5, 33)
Wisconsin	28 (8, 48)	12 (5, 25)
Rhode Island	29 (8, 49)	38 (30, 45)
Nevada	30 (7, 50)	31 (19, 40)
Georgia	33 (13, 48)	37 (28, 45)
Maine	34 (13, 49)	44 (41, 46)
Nebraska	34 (12, 49)	38 (31, 45)
Florida	35 (22, 45)	33 (28, 38)
Illinois	35 (21, 45)	20 (11, 31)
North Carolina	35 (13, 49)	39 (33, 44)
North Dakota	35 (12, 50)	11 (5, 20)
Texas	35 (22, 45)	5 (1, 12)
Mississippi	36 (13, 50)	30 (19, 39)
Washington, DC	37 (11, 51)	–
Indiana	37 (15, 50)	27 (12, 37)
Oklahoma	38 (16, 50)	15 (7, 28)
South Carolina	38 (15, 50)	34 (23, 42)
Arizona	40 (19, 50)	10 (3, 23)
Virginia	40 (20, 50)	44 (38, 46)
Delaware	41 (17, 51)	23 (14, 31)
Minnesota	45 (26, 51)	–
Kentucky	46 (28, 51)	13 (6, 24)
Alaska	47 (28, 51)	43 (37, 46)
Tennessee	47 (32, 51)	30 (19, 38)
Utah	50 (40, 51)	47 (47, 47)

In YRBSS, the prevalence of adolescent binge alcohol drinking ranged from 7.1% (SE, 0.7%) in Utah to 21.7% (SE, 0.5%) in Montana, with a median of 17.1% (Table 2). In paired samples *t *tests, rates of binge alcohol drinking were higher in YRBSS than in NSDUH across states (*P* < .001). Montana was ranked 2 (95% CI, 1-6) and Utah 47 (95% CI, 47-47) in binge alcohol drinking (Figure 2). Again, ranks across the surveys were not necessarily equal, but they were correlated (*r* = 0.36, *P* = .01).

### Marijuana use

**Figure 3:**
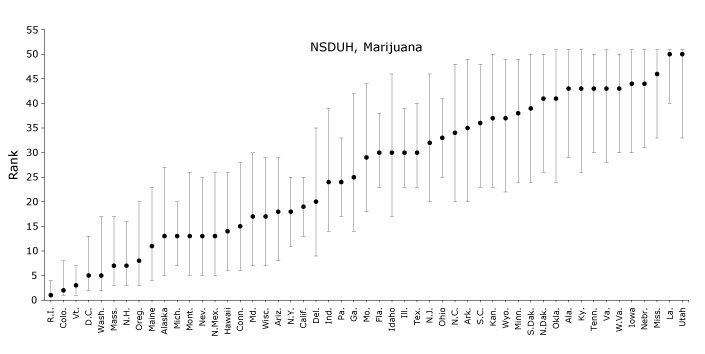

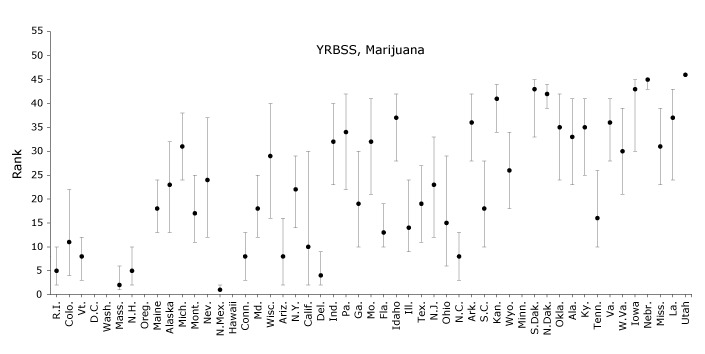
States’ simulated ranks for adolescent (aged 14–17 y) marijuana use as reported in the National Survey on Drug Use and Health (NSDUH) or Youth Risk Behavior Surveillance System (YRBSS), 2011–2015. Past-month marijuana use was defined as using marijuana at least once in the previous 30 days. YRBSS data were collected in 2011, 2013, and 2015; District of Columbia, Minnesota, Oregon, and Washington were excluded from YRBSS because they either chose not to participate or did not achieve adequate response rates. Data on marijuana use in YRBSS were not available for Hawaii. States are ordered by median rank in NSDUH across 100,000 simulations. Error bars indicate 95% confidence intervals. **Table:** 

In NSDUH, the prevalence of adolescent self-reported marijuana use ranged from 6.3% in Louisiana (SE, 0.9%) and Utah (SE, 1.4%) to 18.7% (SE, 1.6%) in Rhode Island, with a median of 9.5% (Table 3). Rhode Island was ranked 1 (95% CI, 1-4) and Louisiana and Utah tied for 50 (95% CI, 40-51, and 33-51, respectively) in adolescent marijuana use (Figure 3). States in the middle of the distribution had wide CIs; for example, Georgia was ranked 25 (95% CI, 14-42).

**Table 3:** Prevalence of Marijuana Use Among Adolescents Aged 14–17 Years and Simulated Ranking of States by Prevalence, National Survey on Drug Use and Health (NSDUH) and Youth Risk Behavior Surveillance System (YRBSS), 2011–2015

State	NSDUH		YRBSS	
	Rank^a^	% (SE)	Rank^a^	% (SE)
Alabama	43	7.6 (0.9)	33	17.1 (0.9)
Alaska	13	12.4 (1.3)	23	18.9 (0.8)
Arizona	18	11.5 (1.1)	8	22.3 (1.2)
Arkansas	35	8.7 (1.2)	36	16.5 (0.7)
California	19	11.3 (0.6)	10	21.5 (2.0)
Colorado	2	16.8 (1.6)	11	21.4 (1.2)
Connecticut	15	12.0 (1.3)	8	22.3 (0.8)
Delaware	20	11.0 (1.2)	4	23.7 (0.8)
District of Columbia	5	14.7 (1.1)	—	—
Florida	30	9.4 (0.5)	13	20.5 (0.5)
Georgia	25	10.0 (1.1)	19	19.6 (1.0)
Hawaii	14	12.2 (1.2)	—	—
Idaho	30	9.4 (1.2)	37	16.3 (0.8)
Illinois	30	9.3 (0.6)	14	20.3 (0.8)
Indiana	24	10.2 (1.0)	32	17.2 (0.9)
Iowa	44	7.5 (1.0)	43	13.8 (1.9)
Kansas	37	8.4 (1.1)	41	15.3 (0.8)
Kentucky	43	7.7 (1.1)	35	16.7 (0.8)
Louisiana	50	6.3 (0.9)	37	16.4 (1.1)
Maine	11	12.8 (1.2)	18	19.6 (0.5)
Maryland	17	11.6 (1.2)	18	19.6 (0.5)
Massachusetts	7	13.9 (1.2)	2	24.6 (0.8)
Michigan	13	12.4 (0.7)	31	17.4 (0.6)
Minnesota	38	8.3 (1.0)	—	—
Mississippi	46	7.2 (0.9)	31	17.3 (0.7)
Missouri	29	9.5 (1.0)	32	17.2 (1.1)
Montana	13	12.4 (1.3)	17	19.8 (0.7)
Nebraska	44	7.5 (0.8)	45	12.5 (0.8)
Nevada	13	12.5 (1.3)	24	18.6 (1.1)
New Hampshire	7	13.9 (1.1)	5	23.2 (0.9)
New Jersey	32	9.1 (1.0)	23	18.9 (1.0)
New Mexico	13	12.5 (1.3)	1	27.1 (1.2)
New York	18	11.4 (0.6)	22	19.0 (0.7)
North Carolina	34	8.9 (1.1)	8	22.3 (0.9)
North Dakota	41	7.9 (1.1)	42	14.5 (0.8)
Ohio	33	9.0 (0.5)	15	20.3 (1.4)
Oklahoma	41	7.9 (1.2)	35	16.6 (1.0)
Oregon	8	13.6 (1.3)	—	—
Pennsylvania	24	10.3 (0.7)	34	17.0 (1.1)
Rhode Island	1	18.7 (1.6)	5	23.3 (0.8)
South Carolina	36	8.6 (0.9)	18	19.7 (0.9)
South Dakota	39	8.2 (1.1)	43	14.2 (1.5)
Tennessee	43	7.7 (0.9)	16	20.0 (0.8)
Texas	30	9.3 (0.6)	19	19.5 (0.8)
Utah	50	6.3 (1.4)	46	8.2 (0.7)
Vermont	3	16.7 (1.4)	8	22.4 (0.7)
Virginia	43	7.7 (1.0)	36	16.6 (0.7)
Washington	5	14.6 (1.5)	—	—
West Virginia	43	7.7 (0.8)	30	17.5 (0.8)
Wisconsin	17	11.7 (1.2)	29	17.8 (1.1)
Wyoming	37	8.5 (1.1)	26	18.2 (0.7)

Abbreviation: SE, standard error.

a Ranks are the median rank generated in 100,000 Monte Carlo simulations. Past-month marijuana use was defined as using marijuana at least once in the previous 30 days. YRBSS data were collected in 2011, 2013, and 2015; District of Columbia, Minnesota, Oregon, and Washington were excluded from YRBSS because they either chose not to participate or did not achieve adequate response rates. Data on marijuana use in YRBSS were not available for Hawaii.

State	NSDUH Rank (95% CI)	YRBSS Rank (95% CI)
Rhode Island	1 (1, 4)	5 (2, 10)
Colorado	2 (1, 8)	11 (4, 22)
Vermont	3 (1, 7)	8 (3, 12)
Washington, DC	5 (2, 13)	–
Washington State	5 (2, 17)	–
Massachusetts	7 (3, 17)	2 (1, 6)
New Hampshire	7 (3, 16)	5 (2, 10)
Oregon	8 (3, 20)	–
Maine	11 (4, 23)	18 (13, 24)
Alaska	13 (5, 27)	23 (13, 32)
Michigan	13 (7, 20)	31 (24, 38)
Montana	13 (5, 26)	17 (11, 25)
Nevada	13 (5, 25)	24 (12, 37)
New Mexico	13 (5, 26)	1 (1, 2)
Hawaii	14 (6, 26)	–
Connecticut	15 (6, 28)	8 (3, 13)
Maryland	17 (7, 30)	18 (12, 25)
Wisconsin	17 (7, 29)	29 (16, 40)
Arizona	18 (8, 29)	8 (2, 16)
New York	18 (11, 25)	22 (14, 29)
California	19 (13, 25)	10 (2, 30)
Delaware	20 (9, 35)	4 (2, 9)
Indiana	24 (14, 39)	32 (23, 40)
Pennsylvania	24 (17, 33)	34 (22, 42)
Georgia	**25 (14, 42)**	19 (10, 30)
Missouri	29 (18, 44)	32 (21, 41)
Florida	30 (23, 38)	13 (10, 19)
Idaho	30 (17, 46)	37 (28, 42)
Illinois	30 (23, 39)	14 (9, 24)
Texas	30 (23, 40)	19 (11, 27)
New Jersey	32 (20, 46)	23(12, 33)
Ohio	33 (25, 41)	15 (6, 29)
North Carolina	34 (20, 48)	8 (3, 13)
Arkansas	35 (20, 49)	36 (28, 42)
South Carolina	36 (23, 48)	18 (10, 28)
Kansas	37 (23, 50)	41 (34, 44)
Wyoming	37 (22, 49)	26 (18, 34)
Minnesota	38 (24, 49)	–
South Dakota	39 (24, 50)	43 (33, 45)
North Dakota	41 (26, 50)	42 (39, 44)
Oklahoma	41 (24, 51)	35 (24, 42)
Alabama	43 (29, 51)	33 (23, 41)
Kentucky	43 (26, 51)	35 (25, 41)
Tennessee	43 (30, 50)	16 (10, 26)
Virginia	43 (28, 51)	36 (28, 41)
West Virginia	43 (30, 50)	30 (21, 39)
Iowa	44 (30, 51)	43 (30, 45)
Nebraska	44 (31, 51)	45 (43, 45)
Mississippi	46 (33, 51)	31 (23, 39)
Louisiana	50 (40, 51)	37 (24, 43)
Utah	50 (33, 51)	46 (46, 46)

In YRBSS, the prevalence of adolescent marijuana use ranged from 8.2% (SE, 0.7%) in Utah to 27.1% (SE, 1.2%) in New Mexico, with a median of 18.9% (Table 3). In paired samples *t* tests, rates of marijuana use were higher in YRBSS than in NSDUH across states (*P* < .001). New Mexico was ranked 1 (95% CI, 1-2) and Utah 46 (95% CI, 46-46) in marijuana use (Figure 3). Again, ranks across the surveys were not necessarily equal, but they were correlated (*r* = 0.69, *P* < .001).

### Correlations of states’ ranks across behaviors

For both surveys, states’ ranks for a given substance use behavior did not correlate highly with their ranks for other behaviors. In NSDUH, the correlation of states’ ranks for cigarette smoking and binge alcohol drinking was 0.24 (*P* = .09), for cigarette smoking and marijuana use, −0.17 (*P* = .23), and for binge alcohol drinking and marijuana use, 0.29 (*P* = .04). In YRBSS, the correlation of states’ ranks for cigarette smoking and binge alcohol drinking was 0.55 (*P* < .001), for cigarette smoking and marijuana use, −0.20 (*P* = .19), and for binge alcohol drinking and marijuana use, −0.01 (*P* = .96).

### Correlation of states’ ranks across subgroups

For both surveys, states’ overall ranks for a given behavior were similar to the ranks derived when examining subgroups of adolescent boys and adolescent girls. In NSDUH, the correlation of states’ overall ranks with ranks for boys was 0.90 for cigarette smoking, 0.66 for binge alcohol drinking, and 0.92 for marijuana use (all *P* < .001). The correlation of overall ranks with ranks for girls was 0.89 for cigarette smoking, 0.83 for binge alcohol drinking, and 0.89 for marijuana use (all *P* < .001). (Indicators and ranks stratified by sex are available from the authors on request.)

Similarly, in YRBSS, the correlation of overall ranks with ranks for boys was 0.95 for cigarette smoking, 0.96 for binge alcohol drinking, and 0.94 for marijuana use (all *P* < .001). The correlation of overall ranks with ranks for girls was 0.96 for cigarette smoking, 0.93 for binge alcohol drinking, and 0.93 for marijuana use (all *P* < .001).

## Discussion

In this analysis, we demonstrated differences in states’ simulated ranks for adolescent substance use across behaviors (ie, cigarette smoking, binge alcohol drinking, marijuana use) and surveys (ie, NSDUH, YRBSS). These findings highlight the variability that emerges when ranking states on behavioral indicators, partly due to differences in behaviors, variation in surveys, and the inherent uncertainty in the statistical ranking processes.

States ranked high on one adolescent substance use behavior did not necessarily rank high on another behavior, reflecting the distinct patterns and correlates of these behaviors. Correlation coefficients between states’ ranks of different behaviors ranged from −0.17 to 0.29 for NSDUH and from −0.20 to 0.55 for YRBSS. Partly, these differences could be attributable to demographic differences across states. For example, adolescent cigarette smoking and alcohol use are inversely associated with parental socioeconomic status (SES), whereas marijuana use has an inverse U-shaped association with SES; further, these effects are moderated by race/ethnicity (18). A more contextual explanation might focus on state policies that influence these behaviors. States’ policies regulating cigarettes include indoor smoke-free air laws, tobacco taxes, and minimum age purchase of cigarettes, all of which could influence adolescent smoking prevalence (7,19,20). Similarly, emerging policies allowing marijuana use (among adults) may be associated with increased adolescent marijuana use (21). Descriptively, in the current analysis, states that have legalized marijuana tended to have higher estimates of adolescent marijuana use. State-level differences in adolescent substance use could also be related to local norms (22,23) or exposure to mass media campaigns discouraging substance use (24).

Across surveys, states’ estimates and ranks for adolescent substance use were not consistent. First, states’ substance use estimates were up to 2.6 times as high in YRBSS as in NSDUH. In addition, the median substance use estimates in NSDUH were 9.3% to 9.5%, whereas the median estimates in YRBSS were 12.5% to 18.9%, indicating greater variability in substance use in YRBSS than NSDUH. Differences in survey administration mode, sampling frame, and item wording could explain this variability in prevalence estimates (25). In terms of administration, NSDUH is delivered in homes (15) and YRBSS in schools (16); for both surveys, figures of authority may be nearby while the survey is administered (parents or teachers, respectively), which could lead to underreporting (8), but adolescents may overreport substance use in YRBSS to impress their peers. However, one recent study of adolescent self-report of substance use found that survey setting did not introduce bias to responses (26). In terms of sampling frame, NSDUH includes (and YRBSS excludes) adolescents who drop out of school, who in turn have higher levels of substance use (25). In terms of item wording, slight differences could account for some differences in estimates; for example, the cigarette smoking item in NSDUH was “During the past 30 days, have you smoked part or all of a cigarette?” (13) whereas the item in YRBSS was “During the past 30 days, on how many days did you smoke cigarettes?” (14). However, efforts to obtain substance use estimates without using self-report on surveys (eg, through biometric tests) would be burdensome to collect in a population-based survey, and a previous study demonstrated construct validity of self-reported adolescent substance use estimates (27). Further, wide differences in states’ estimates of adolescent substance use translated into wide differences in states’ ranks across surveys. When comparing ranks in NSDUH versus YRBSS, the correlations were 0.64 for cigarette smoking, 0.36 for binge alcohol drinking, and 0.69 for marijuana use. The overall inconsistency is depicted in the YRBSS panels of Figures 1, 2, and 3, which are organized by NSDUH rank and appear fairly scattered. These findings indicate that the biases inflating YRBSS estimates compared with NSDUH estimates do not operate similarly across states; if they did, we would expect to see similar patterns of ranks across surveys. Overall, the discrepancies between estimates and ranks across surveys suggest that biases in data collection exist, which could have important implications for policy setting and intervention development.

Finally, the statistical process of ranking states while accounting for variability in the underlying estimates of adolescent substance use revealed a lack of precision. The ranks’ 95% CIs were wide, especially for states in the middle of each distribution, where CIs spanned 20 or more ranks. Although the first- and last-ranked states were statistically distinguishable, many of the states between the extremes had overlapping CIs. The research literature on ranking states (or other geographic jurisdictions, such as counties) has been engaged in a debate as to whether ranks are “good enough” to present without measures of error (11,28–30), but our findings suggest such a practice overstates the precision of ranks. Presenting ranks without measures of error may be more digestible to the public and to policy makers, but it could lead to overinterpretation of the ranks and misappropriation of public health funding.

Despite these differences, some consistent findings support conclusions about substance use among adolescents across states. Utah consistently ranked last across behaviors and surveys for adolescent substance use, reflecting the relatively low proportions of adolescents engaging in these behaviors. This finding could be related to Utah’s high levels of religiosity (31) — a construct that has been linked to lower prevalence of adolescent substance use (19,32) — and restrictions on the sale and use of the substances under study (21,33). To the extent possible, adapting programs and policies from Utah (or other well-performing states) for adoption in other jurisdictions could reduce the prevalence of adolescent substance use. In addition, we found that correlations of states’ ranks for their overall population with the ranks derived for boys or girls only (0.66–0.96) were similar. Thus, state processes affecting self-reported adolescent substance use appear to do so for boys and girls similarly.

Taken together, these findings have implications for cancer prevention. Given that adolescent substance use prevalence estimates and rankings varied across surveys and behaviors, identifying “high-need” states for additional research or interventions is difficult. That is, a state that ranked poorly for cigarette smoking on one survey did not necessarily rank poorly on the other survey, and it did not necessarily rank poorly for other outcomes. Thus, selecting high-need states for behavior-specific research depends on the survey used, and selecting high-need states for research on multiple substance use behaviors requires care. However, calculating 95% CIs around ranks affords some flexibility because it allows researchers not only to recognize the uncertainty in rankings but also to identify states that have moderate ranks whose CIs include the poorest ranks. For example, Wyoming is ranked 1 and 4 for cigarette smoking and 12 and 5 for binge alcohol drinking in NSDUH and YRBSS, respectively, but its 95% CIs all include at least rank 2, indicating that it is in the top 5% of states in terms of adolescent use of these 2 substances (a pattern that would not have been immediately discernible without 95% CIs). From the perspective of states that performed well on all indicators, Utah was ranked last (indicating low adolescent substance use prevalence) on all behaviors, which could offer some clues for cancer prevention activities. Overall, however, these findings underscore the need for additional research in at least 2 areas: 1) improving surveillance of cancer prevention behaviors, especially as prevalence estimates appear to be sensitive to survey mode, and uncertainty in the state rankings was evident; and 2) hypothesis generation and testing for state characteristics, programs, and policies that can discourage adolescent substance use for the purpose of lifelong cancer prevention.

This study has several limitations. NSDUH and YRBSS both used self-reported measures of substance use, which are subject to biases (8). Not all states participated in YRBSS (16), restricting the range of ranks and the scope of our inferences. However, NSDUH collected data for all states, allowing us to examine the entire United States. In 2015, NSDUH changed their definition of binge drinking for adolescent girls (15), so the estimates of binge drinking included data only from 2011–2014. Finally, we did not examine the emerging use of other substances that might be related to cancer risk among adolescents, such as e-cigarettes (34). In terms of study strengths, our analysis leveraged data from more than half a million participants in 2 nationally representative surveys using different administration modes. This generous sample size allowed us to produce stable state-level estimates of adolescent substance use. Finally, our research points to the need for improved methodology to rank and compare states’ performance on public health indicators.

Adolescent substance use that contributes to cancer risk is relatively common (4.5% to 27.1% across surveys and behaviors). In 2 population-based surveys, we found some consistency in performance for selected states and across subgroups. However, great variability emerged in states’ rankings, potentially due to differences in behaviors, survey methods, and statistical procedures. Generally, we could not distinguish among states’ performance on adolescent substance use with certainty. Yet public health officials may be able to adopt policies and programs in states that had low estimates of substance use (eg, Utah) to reduce adolescent cigarette smoking, binge alcohol drinking, and marijuana use elsewhere. Such a goal is important for reducing morbidity and mortality among adolescents now (eg, from vehicular crashes when the driver is under the influence of alcohol) and as they grow older (eg, from cancers associated with cigarette, alcohol, and marijuana use).
